# Bioelectrical Impedance Analysis Reveals Altered Hydration and Body Composition Profiles in Patients With Primary Ciliary Dyskinesia

**DOI:** 10.7759/cureus.109984

**Published:** 2026-05-31

**Authors:** Ian M Santiago Velazquez, Karina L Rivera García, Natalia M Ortiz Pérez, Wilfredo De Jesús-Rojas

**Affiliations:** 1 Basic Sciences, Ponce Health Sciences University, Ponce, PRI; 2 Pediatrics, Ponce Health Sciences University, Ponce, PRI

**Keywords:** bioelectrical impedance measurement, body composition, fev 1, lung transplantation candidacy, primary ciliary dyskinesia (pcd), pulmonary function

## Abstract

Background: Primary ciliary dyskinesia (PCD) is a rare inherited disorder that can progress to advanced lung disease requiring lung transplantation. Current assessment strategies rely primarily on pulmonary function and imaging, which may not adequately capture systemic physiologic reserve or overall fitness relevant to transplant candidacy. Body composition analysis has emerged as an important marker of functional status in chronic lung diseases, yet its role in PCD remains poorly defined.

Methods: We conducted an observational cross-sectional study to characterize body composition in 16 patients with PCD due to the *RSPH4A* founder variant undergoing baseline evaluation for potential lung transplantation. Body composition was assessed using multi-frequency segmental bioelectrical impedance analysis (BIA) (MC-780U PLUS; Tanita Corporation, Tokyo, Japan), including measurements of body mass index (BMI), skeletal muscle mass, body fat percentage (Fat%), visceral fat rating (VFR), total body water percentage (TBW%), and extracellular-to-total body water ratio (ECW/TBW). Spirometric data, including forced expiratory volume in one second (FEV₁% predicted), were available for all participants. Descriptive statistics were generated, and relationships between body composition parameters and lung function were explored using Spearman rank correlation analysis.

Results: Median BMI in the cohort was 21.35 kg/m² (IQR 19.63-28.48), while median TBW% was 51.55%. Compared with individualized target values, TBW% was consistently lower, and ECW/TBW ratios were higher, suggesting altered body fluid distribution in patients with PCD. Muscle mass and fat measures demonstrated notable inter-individual variability. Despite these physiologic alterations, no statistically significant correlations were identified between FEV₁% and body composition parameters, including Fat%, age, TBW%, BMI, ECW/TBW, and muscle mass. Spearman correlation coefficients ranged from -0.26 to 0.22, with all p-values exceeding 0.31 at a significance threshold of α = 0.05. Age-stratified analyses suggested greater variability in BMI and fat-related measures among patients ≥25 years.

Conclusions: In patients with PCD due to the *RSPH4A* variant (n=16), body composition analysis using multi-frequency BIA demonstrated reduced TBW% and elevated ECW/TBW, suggesting altered hydration status. Heterogeneous muscle and fat distribution was also observed, suggesting variability in body composition not fully captured by BMI. No transplant-related outcomes were assessed, and therefore clinical applicability to transplant evaluation cannot be determined. Multi-frequency BIA may provide a noninvasive method to characterize body composition, but larger longitudinal studies are required to determine associations with disease progression, functional decline, and transplant outcomes in these patients.

## Introduction

Primary ciliary dyskinesia (PCD) is a rare, inherited disorder characterized by dysfunction of motile cilia, leading to impaired mucociliary clearance, chronic airway infection, and progressive bronchiectasis [1]. Over time, a subset of patients develops advanced lung disease requiring consideration for lung transplantation [1]. While disease monitoring in PCD has traditionally focused on pulmonary function testing and imaging, these approaches primarily capture airway-centered pathology and may fail to reflect systemic physiologic impairment or overall fitness relevant to lung transplant candidacy [2]. In chronic respiratory diseases, body composition has emerged as a critical determinant of clinical outcomes [3]. Alterations in skeletal muscle mass, fat, and body water distribution are increasingly recognized as markers of functional capacity, frailty, and prognosis [4]. In conditions such as cystic fibrosis and chronic obstructive pulmonary disease (COPD), reduced fat-free mass and sarcopenia are associated with decreased exercise tolerance, increased hospitalization, and worse survival, independent of lung function [5]. Similarly, abnormalities in fluid distribution, including elevated extracellular-to-total body water ratio (ECW/TBW), have been linked to systemic inflammation and adverse outcomes in advanced pulmonary disease and transplant populations [6].

Despite these insights, the role of body composition in PCD remains poorly defined [2]. Given the chronic inflammatory burden, recurrent infections, and increased work of breathing characteristic of PCD, patients may be particularly susceptible to alterations in muscle mass and fluid balance [1]. However, current clinical paradigms in PCD remain largely lung-centric and do not routinely incorporate assessments of systemic physiologic reserve [2]. This represents a critical knowledge gap, particularly in patients being evaluated for lung transplantation, where nutritional status, muscle mass, and fluid balance are known to influence perioperative risk and post-transplant outcomes in these populations [7]. To address this gap, we conducted an observational cross-sectional study to characterize body composition in patients with PCD using multi-frequency bioelectrical impedance analysis (BIA) [8]. Therefore, body composition assessment may provide complementary physiologic characterization beyond spirometric measures in patients with PCD with advanced lung disease who are evaluated in lung transplant settings.

We hypothesized that patients with PCD will exhibit alterations in water distribution, including a reduced total body water percentage (TBW%) and an elevated ECW/TBW, as well as significant inter-individual variability in muscle and fat compartments. We conducted an observational cross-sectional study with the objective of characterizing body composition in patients with PCD due to the *RSPH4A* founder variant, including BMI, muscle mass, Fat%, TBW%, and ECW/TBW, with an emphasis on hydration status, skeletal muscle mass, and fat distribution patterns, evaluating the association between body composition parameters and lung function (forced expiratory volume in one second (FEV₁) predicted), and exploring the potential relevance of these measures to lung transplant candidacy.

Prior studies have demonstrated associations between bioelectrical impedance-derived parameters and lung function in chronic respiratory diseases such as COPD [9,10], further supporting the rationale for examining these relationships in patients with PCD.

## Materials and methods

Study design and population

This observational, cross-sectional study evaluated body composition in 16 patients with PCD due to the *RSPH4A* founder variant who were undergoing baseline assessment for potential lung transplantation. The study included three male and 13 female participants, with a median age of 30 years (IQR: 21-57). Age stratification included seven participants <25 years and nine participants ≥25 years. All participants were evaluated in a specialized pulmonary clinic setting in Puerto Rico as part of routine clinical care. Measurements were performed after an overnight fast, with patients having voided within 30 minutes, and no strenuous exercise for 12 hours prior. No diuretic, steroid use, or strenuous exercise within 12 hours prior to assessment was reported. All patients denied a history of renal disease. Demographic variables collected included age and sex. Clinical data were obtained at a single time point corresponding to the transplant evaluation visit. Inclusion criteria consisted of genetically confirmed PCD due to the *RSPH4A* genetic variant and availability of both body composition and spirometry data. Given the exploratory nature of the study and the rarity of the condition, all eligible patients during the study period were included. The study protocol was approved by the Institutional Review Board of the Ponce Medical School Foundation, Inc. (approval number: 2512299384), and all procedures were conducted in accordance with institutional ethical standards.

Pulmonary function assessment

Pulmonary function data were obtained from spirometry performed according to the American Thoracic Society (ATS) and European Respiratory Society (ERS) standardized guidelines. Consensus for general considerations extracted from the International Society for Heart and Lung Transplantation is listed in Table 1. Measurements were conducted using calibrated equipment by trained personnel, ensuring acceptability and reproducibility criteria were met. Forced expiratory volume in one second (FEV₁) was recorded and expressed as percent predicted (FEV₁%), based on reference equations appropriate for age, sex, height, and ethnicity. Spirometry data were available for all participants (n=16) and were used to assess the relationship between lung function and body composition parameters.

**Table 1:** Summary of key pre-transplant clinical factors influencing candidate selection and outcomes in lung transplantation Although this information was extracted from the Consensus document for the selection of lung transplant candidates published by the International Society for Heart and Lung Transplantation [11], it does not encompass the complete set of criteria and considerations for lung transplantation assessment described in the original source. Albumin is reported in grams per deciliter (g/dL).

Domain	Key Considerations	Clinical Implications
Age	Increasingly accepted in older candidates	Associated with reduced long-term survival; ethical considerations regarding allocation of scarce organs
Malignancy	Requires thorough screening; must exclude active or metastatic disease	High-risk malignancy is an absolute contraindication; risk varies by cancer type
Renal Function	Risk of post-transplant renal failure	Need for renal replacement therapy is associated with worse outcomes
Coronary Artery Disease	Mild–moderate coronary artery disease or revascularized disease	Does not significantly worsen survival in selected patients
Peripheral Vascular Disease	Marker of systemic atherosclerosis and comorbidity	Important in overall risk assessment; no clear exclusion threshold
Heart Failure	Right heart failure acceptable; limited data on left ventricular dysfunction	Low left ventricular ejection fraction often considered a contraindication at many centers
Connective Tissue Disease	Careful patient selection required	Comparable survival and graft outcomes to other indications
Esophageal Dysfunction/Gastroesophageal Reflux Disease	Associated with reflux and dysmotility	Linked to acute rejection, infection, and chronic lung allograft dysfunction; anti-reflux surgery may improve outcomes
Hematologic Abnormalities	Includes cytopenias, coagulopathies, bone marrow dysfunction	May increase perioperative risk and limit immunosuppression; severe cases are high-risk
Body Mass Index	Both high and low body mass index impact outcomes	Obesity linked to graft dysfunction and mortality; body mass index is an imperfect surrogate for body composition
Hypoalbuminemia	Albumin <3.5 g/dL	Associated with decreased survival and increased postoperative complications
Functional Status and Frailty	No standardized assessment tools	Functional status predicts outcomes; frailty interpretation should be cautious
Human Leukocyte Antigens Sensitization	Presence of donor-specific antibodies	Increases difficulty in donor matching and risk of poor outcomes; variability in desensitization practices

Body composition assessment

Body composition was assessed using multi-frequency segmental BIA with the MC-780U PLUS Multi-Frequency Segmental Body Composition Analyzer (Tanita Corporation, Tokyo, Japan). This device measures impedance at six frequencies across five body segments (trunk and four limbs) to estimate whole-body and segmental composition parameters. Impedance measurements were obtained using hand and foot electrodes integrated into the device, enabling segmental assessment of the trunk and extremities.

**Figure 1:**
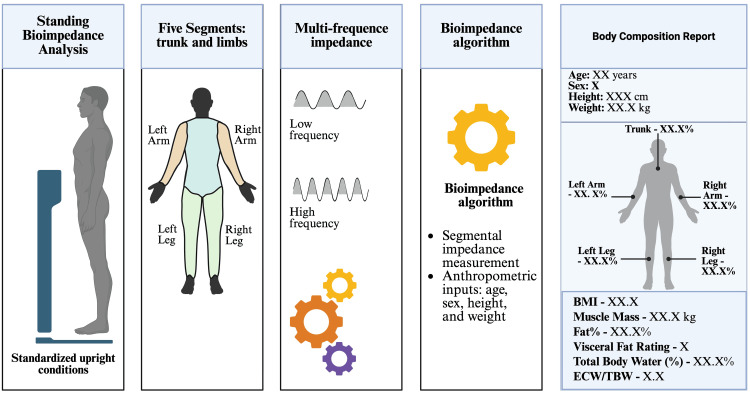
Workflow of multi-frequency segmental bioelectrical impedance analysis using the Tanita MC-780U System This table illustrates the process of obtaining a body composition report from a multi-frequency segmental bioelectrical impedance analysis using the Tanita MC-780U System. BMI: body mass index, ECW/TBW: extracellular water to total body water ratio Units are expressed as kilograms (kg); kilocalories per day (kcal/day); percentage (%), or years, as appropriate. Segmental percentages illustrated in the right-hand-side image (Trunk, Left Arm, Left Leg, Right Arm, and Right Leg) represent fat percentage (Fat%) distribution of the patient. The figure was created by the authors using BioRender (BioRender, Ontario, Canada), and no artificial intelligence (AI) tools were used in its creation.

Measurements were performed under standardized conditions, with participants evaluated in an upright position according to manufacturer recommendations. The manufacturer’s proprietary BIA algorithm incorporates impedance-derived estimates with anthropometric variables (age, sex, height, and weight) to calculate body composition metrics. Although all device-generated variables were recorded, primary analyses focused on parameters relevant to physiologic reserve and transplant candidacy, including BMI, total skeletal muscle mass, body fat percentage, visceral fat rating (VFR), TBW%, and ECW/TBW. These parameters were selected based on their established associations with functional status, inflammation, and outcomes in chronic pulmonary disease and transplant populations. All body composition parameters included in the analysis and their corresponding units are summarized in Table 2. The principles of multi-frequency segmental BIA and device workflow are illustrated in Figure 1.

**Table 2:** Body composition variables and units This table illustrates the body composition variables along with their corresponding units. BMI: body mass index, VFR: visceral fat rating, ECW: extracellular water, ICW: intracellular water, ECW/TBW: extracellular water to total body water ratio, TBW: total body water, BMR: basal metabolic rate Units are expressed as kilograms (kg), kilocalories per day (kcal/day), percentage (%), or years, as appropriate.

Parameter	Unit
Weight	kg
BMI	kg/m^2^
Fat%	%
VFR	unitless
Fat Mass	kg
Fat-Free Mass	kg
Muscle Mass	kg
TBW	kg
TBW%	%
ECW	kg
ICW	kg
ECW/TBW	ratio
Basal Metabolic Rate (BMR)	kcal/day
Metabolic Age	years

Reference and target value determination

For each body composition parameter, deviations from individualized target values were calculated using the formula: Δ = measured - target. Positive values indicated measurements above target, whereas negative values indicated measurements below target. Device-provided individualized target values were derived from manufacturer-specific reference algorithms that account for age, sex, and body size. To enable cohort-level visualization of expected values, they were normalized to a 0-100 scale. Because the Tanita MC-780U System report does not provide individualized hydration status, expected values were estimated using the Watson formula, which incorporates age, sex, height, and weight as an external estimate of reference TBW% per patient. The median measured TBW by BIA was 51.55% (IQR: 45.38-54.73), which was lower than the median TBW of 62.1% (IQR: 57.73-73.72) predicted by the Watson formula. This provided an external physiologic benchmark to contextualize device-derived TBW measurements and to assess potential alterations in body fluid distribution relative to target values.

Data normalization and visualization

To facilitate comparative visualization across multiple physiologic domains, selected body composition variables were normalized for radar plot representation. Each measured value was expressed as a proportion of its corresponding individualized target value and subsequently scaled to a standardized 0-100 range. Within this framework, target values were fixed at 50 to represent the reference midpoint, while measured values were scaled proportionally above or below this point according to their percentage deviation from target. This approach allowed for intuitive visualization of relative deficits or excesses across parameters within individual patients and across subgroups.

Statistical analysis and stratified analyses

Descriptive statistics were calculated to summarize the distribution of body composition and pulmonary function variables. These included medians, interquartile ranges (IQR), minimum and maximum values, ranges, and median absolute deviation normalized to the median (MAD/median), providing a robust measure of variability less sensitive to outliers. All descriptive analyses were performed using Microsoft Excel (Microsoft Corporation, Redmond, WA, USA). Comparisons between measured and individualized target values were conducted using the Wilcoxon signed-rank test, given the paired nature of the data and non-parametric distribution assumptions. Associations between body composition parameters and pulmonary function (FEV₁%) were evaluated using Spearman’s rank correlation coefficients. All inferential statistical analyses were performed using GraphPad Prism (GraphPad Software, San Diego, CA, USA), with a two-sided significance threshold of α=0.05. Given the exploratory design and small sample size, no adjustments were made for multiple comparisons. To explore potential age-related differences in body composition, analyses were stratified by age (<25 years vs ≥25 years). These thresholds were selected to approximate transitions in adult physiology and potential cumulative disease burden. Comparisons across age groups focused on variability and distribution of key parameters, including BMI, Fat%, muscle mass, and fluid-related indices (TBW% and ECW/TBW). Due to limited sample size, these analyses were considered descriptive and hypothesis-generating. Individual patient-level body composition and pulmonary function data are presented in Table 3.

**Table 3:** Individual patient body composition parameters and pulmonary function (FEV₁%) This table illustrates various body composition parameters and pulmonary function. BMI: body mass index, VFR: visceral fat rating, TBW: total body water, TBW%: total body water percentage, ECW: extracellular water, ICW: intracellular water, ECW/TBW: extracellular water to total body water ratio, BMR: basal metabolic rate, FEV₁%: forced expiratory volume in one second Units are expressed as Kilograms (kg), percentage (%), and kilocalories (kcal), as appropriate.

Patient	Weight (kg)	BMI	Fat%	VFR	Fat Mass	Muscle Mass	TBW (kg)	TBW%	ECW	ICW (kg)	ECW/TBW	BMR (kcal)	Metabolic Age	FEV₁ (%)
1	45.18	17.1	18.7	1	18.6	76.8	26.77	60.1	23.6	16.45	0.395	1158	12	65
2	60.51	19.1	25.6	1	34.2	94.2	30.27	49.9	29.4	16.91	0.440	1378	19	49
3	74.66	25.8	31.6	4	52.0	106.8	34.27	45.8	33.8	18.91	0.447	1533	39	51
4	71.58	27.1	36.1	4	56.8	95.6	31.55	44.1	32.4	16.82	0.467	1464	34	39
5	81.01	32.7	37.8	8	67.4	105.4	34.64	42.7	36.2	18.27	0.474	1531	57	55
6	92.08	35.9	45.4	13	92.2	105.2	35.27	38.3	38.8	17.64	0.500	1547	77	58
7	52.80	20.0	26.5	1	30.8	81.0	28.73	54.5	25.8	17.00	0.408	1229	15	56
8	52.44	19.8	16.3	2	18.8	91.8	34.09	64.9	31.4	19.82	0.418	1339	12	65
9	100.15	32.6	28.8	15	63.4	149.4	51.73	51.5	45.6	31.00	0.401	2102	66	49
10	61.05	26.3	39.4	5	53.0	77.4	29.55	48.4	27.2	17.27	0.417	1204	40	51
11	86.64	41.4	43.1	13	82.2	103.2	36.45	42.0	37.2	19.55	0.464	1510	72	63
12	65.05	21.2	25.8	5	37.0	100.8	36.09	55.4	34.6	20.36	0.436	1482	36	38
13	58.69	21.5	19.4	2	25.2	98.8	31.64	53.9	29.4	18.36	0.421	1376	26	77
14	46.90	18.3	17.2	7	17.8	81.2	28.82	61.4	30.2	15.09	0.476	1127	48	23
15	54.34	21.2	20.7	4	24.6	90.2	28.27	51.9	28.2	15.45	0.452	1247	47	37
16	57.61	18.2	14.5	1	18.6	103.0	29.73	51.6	30.4	15.91	0.465	1477	12	61

## Results

Table 4 displays the distribution of body composition parameters in this cohort (n=16). Variables are reported using the five-number summary, range, and MAD/median. MAD/median ratio is a scale-independent measurement of relative dispersion from the population's median. Higher MAD/median values indicate greater heterogeneity across the cohort, while lower values indicate greater homogeneity around the median.

**Table 4:** Distribution of measured body composition parameters in the PCD cohort This table illustrates the distribution of the body composition parameters of patients with PCD-*RSPH4A* who were evaluated in an outpatient clinic using bioimpedance analysis. Min: minimum, Q1: first quartile, median, Q3: third quartile, Max: maximum, IQR: interquartile range, and MAD/median: mean of absolute deviation to median ratio. Parameters used in this table include muscle mass, TBW%: total body water percentage, ECW/TBW: extracellular water to total body water ratio, Fat%: fat percentage, and BMI: body mass index

Parameter	Min	Q1	Median	Q3	Max	IQR	Range	MAD/Median
Muscle mass	76.80	87.95	97.20	103.70	149.40	15.75	61.45	0.13
TBW%	38.30	45.38	51.55	54.73	64.90	9.35	19.53	0.13
ECW/TBW	0.40	0.42	0.44	0.47	0.5	0.048	0.08	0.06
Fat%	14.50	19.23	26.15	36.53	45.40	17.30	26.18	0.31
BMI	17.10	19.63	21.35	28.48	41.40	8.85	21.78	0.28

MAD/median shows greater heterogeneity in Fat% and BMI, reflecting higher dispersion around the median for these parameters compared with the remaining parameters.

Table 4 summarizes the measured body composition parameters for the cohort, and Table 5 presents the corresponding deviations from patient-specific target values. For each patient, measured parameters were compared with target values derived from BIA, and the resulting differences were used to characterize deviations from expected outcomes. Positive values indicate measurements above the target, whereas negative values indicate measurements below the target.

**Table 5:** Deviations of measured body composition parameters from patient-specific targets in the PCD cohort provided by BIA This table illustrates the deviations between measured body composition values and patient-specific desirable targets (Δ = measured - target) for patients with PCD-*RSPH4A* (n=16). All variables are reported using Min = Minimum, Q1 = First quartile, Median, Q3 = Third quartile, and Max = Maximum. Parameters used in this table include muscle mass, TBW%: total body water percentage, ECW/TBW: extracellular water to total body water ratio, Fat%: fat percentage, and BMI: body mass index

Parameter	Min	Q1	Median	Q3	Max
Muscle mass	4.60	-0.2	4.65	-5.2	19.45
TBW%	-14.62	-12.35	-10.53	-18.99	-26.78
ECW/TBW	0.02	0.04	0.07	0.09	0.13
Fat%	0.55	-4.6	-0.8	8.08	15.45
BMI	-4.60	-2.08	-0.35	6.78	19.70

Across the cohort, Table 5 shows negative deviations for TBW% and positive deviations for ECW/TBW, suggesting a relative reduction in TBW% and increases in ECW/TBW compared with the target. These deviations in TBW% (p = 0.0021) and ECW/TBW (p < 0.0001) were statistically significant as evidenced by the results of the Wilcoxon signed-rank test. All other deviations observed in Table 5, including muscle mass (p = 0.8209), Fat% (p = 0.1624), and BMI (p = 0.2798), were not found to be statistically significant deviations.

**Figure 2:**
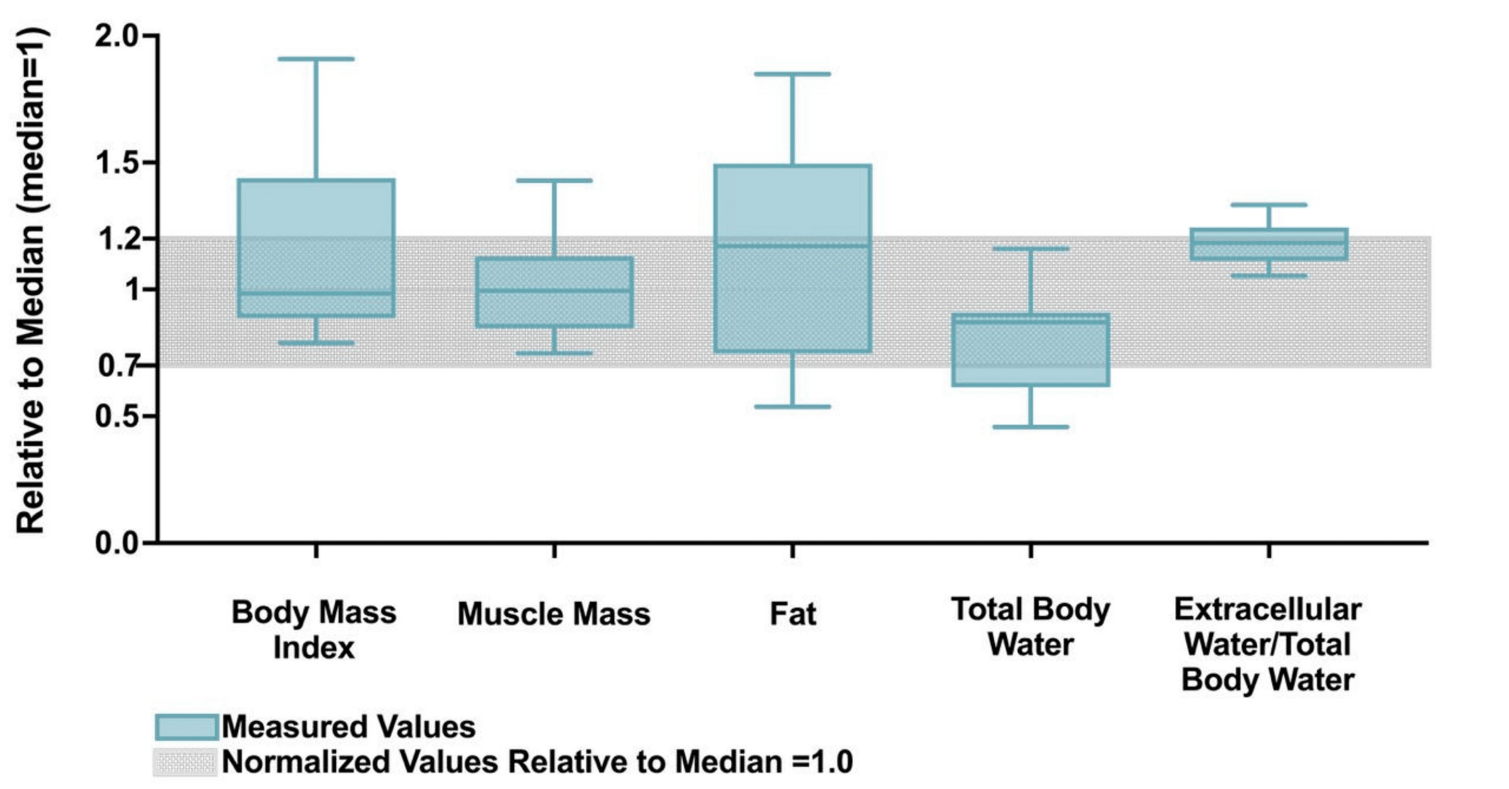
Relative distribution of measured body composition parameters in the PCD cohort This figure illustrates boxplots of five body composition parameters measured by bioimpedance analysis in patients with PCD-*RSPH4A* (n=16). Values are expressed relative to the median to enable comparison of dispersion across parameters with different units and scales. The boxes display the interquartile range; the light grey rectangle designates the normalized target range; and 1 indicates the cohort median. PCD: primary ciliary dyskinesia The figure was created by the authors using GraphPad Prism (GraphPad Prism, San Diego, California).

Figure 2 displays a normalized visualization of the values that were reported in Table 3, generated using a measured-to-target ratio (measured/target), with the target value standardized to 1. A reference range of 0.7-1.2 was defined based on BIA-derived target values. VFR was excluded because no corresponding target value was available in the BIA dataset report.

In Figure 2, most values (muscle mass, TBW%, and ECW/TBW) fall below the defined normal range depicted by the light grey rectangle between 7 and 1.2, while BMI and Fat% show greater dispersal, consistent with the higher MAD/median ratios reported in Table 4.

**Figure 3:**
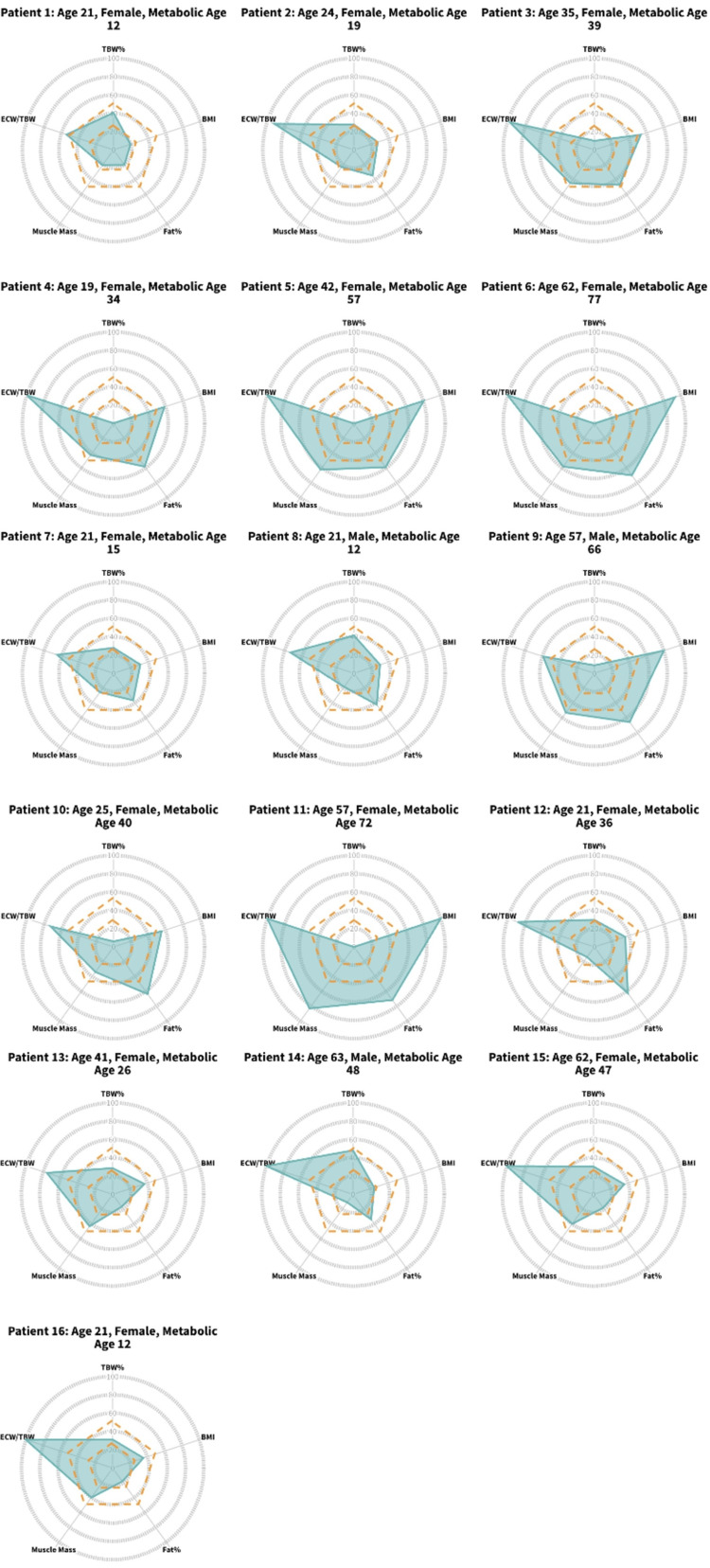
Patient-level body composition profiles relative to target values in the PCD cohort This figure presents radar plots of patient-level body composition profiles for PCD-*RSPH4A* (n=16). Each plot corresponds to a single patient and displays demographic information, including sex, chronological age, and metabolic age. Five parameters are shown: BMI: body mass index, muscle mass, Fat%: fat percentage, TBW%: total body water percentage, and ECW/TBW: extracellular water to total body water ratio. All parameters normalized to a 0-100 scale to facilitate within-patient and between-patient comparisons across variables with different units. The solid teal polygon represents measured values, while the dashed orange unfilled polygon denotes patient-specific desirable target values. PCD: primary ciliary dyskinesia The figure was created by the authors using Flourish Studio (Flourish, London, UK).

All variables were visualized using radar plots (Figure 3) to illustrate each patient’s profile relative to BIA-derived target values. Measured values were rescaled to a 0-100 scale for visualization purposes. Target values were mapped to a reference band between 25 and 50 to indicate the target acceptable range.

Across the cohort, hydration parameters (TBW% and ECW/TBW) and fat distribution (Fat%) exhibit variation from the target range of 25-50, consistent with the patterns observed in Table 5 and Figure 2.

Statistical comparisons between measured and target values for TBW%, BMI, Fat%, muscle mass, and ECW/TBW were performed using the Wilcoxon signed-rank test, with a significance threshold of α=0.05. Analyses were stratified by age.

For participants aged >25 years, muscle mass (p=0.1641), TBW% (p=0.0195), ECW/TBW (p=0.0039), Fat% (p=0.0977), and BMI (p=0.0309) were analyzed.

For participants aged <25 years, muscle mass (p=0.0312), TBW% (p=0.1016), ECW/TBW (p=0.0156), Fat% (p>0.999), and BMI (p=0.2969) were analyzed.

Overall, muscle mass (p=0.8209), TBW% (p=0.0021), ECW/TBW (p<0.0001), Fat% (p=0.1624), and BMI (p=0.2798) were analyzed.

**Figure 4:**
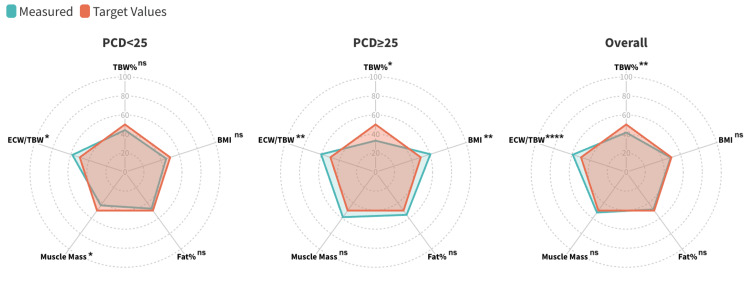
Median body composition analysis for median versus target median comparison for PCD cohort This figure illustrates the median body composition profiles in the PCD-*RSPH4A* (n=16) stratified by age (<25 years and ≥25 years). Age stratification included seven participants <25 years and nine participants ≥25 years. Radar plots display a normalized 0-100 scale of median body composition variables. TBW%: total body water percentage, BMI: body mass index, Fat%: fat percentage, muscle mass, and ECW/TBW: extracellular water to total body water; PCD: primary ciliary dyskinesia Measured median values are displayed in the solid teal polygon, while desirable targets are indicated by the orange polygon at the 50 mark. With a significance threshold at α=0.05, parameters marked with * indicate statistically significant differences (p<0.05), ** indicate p<0.01, *** indicate p<0.001, and ns denotes no statistically significant difference. The figure was created by the authors using Flourish Studio (Flourish, London, UK).

Figure 4 illustrates patient profiles stratified by age, with 25 years as the cutoff to distinguish pediatric and adult patients with PCD-*RSPH4A*, presented as radar plots.

In Figure 4, hydration parameters (ECW/TBW and TBW%) show statistically significant deviations from target values, with ECW/TBW exceeding and TBW% falling below the desired values. Age-stratified analysis indicates that ECW/TBW and muscle mass are significantly different from target values in patients under 25, whereas TBW%, ECW/TBW, and BMI are significantly different in patients aged 25 and older. Additionally, patients in the older group have higher BMIs than target values.

## Discussion

This cross-sectional study examined body composition in patients with the PCD-*RSPH4A* variant evaluated in an outpatient clinical setting. All 16 patients were previously diagnosed with PCD due to the *RSPH4A* founder variant (c.921+3_921+6delAAGT), which is prevalent in the Hispanic population [12]. Among the 16 patients included in the study, both hydration parameters, TBW% and ECW/TBW, showed consistent deviations from individualized targets, with lower TBW% and elevated ECW/TBW, suggesting altered fluid distribution (Figure 4). All BIA data were obtained after an overnight fast, with patients having voided within 30 minutes. No diuretic, steroid use, or strenuous exercise within 12 hours prior to assessment was reported. All patients denied a history of renal disease. Results must be used with caution due to the device's proprietary algorithms and may not be reproducible across BIA devices.

In PCD, recurrent infections and ineffective mucus clearance lead to bronchiectasis, persistent inflammation, and systemic physiologic stress, all of which may contribute to alterations in body composition and fluid balance [1]. These changes are clinically relevant because they can affect the precision and accuracy of BIA measurements [13]. Although data specific to PCD remain limited, similar alterations in extracellular water distribution have been described in COPD, where fluid distribution measures are associated with increased symptom burden, diminished pulmonary function, and more severe disease [9]. These findings may reflect shared mechanisms of chronic airway inflammation and impaired mucociliary clearance [1]. Despite these observations, no statistically significant associations were identified between FEV₁% and body composition parameters, including Fat%, age, TBW%, BMI, ECW/TBW, and muscle mass in this cohort. Spearman correlation coefficients ranged from -0.26 to 0.22, with all p-values exceeding 0.30 at a significance threshold of α=0.05.

Several studies have evaluated the relationship between body composition and lung function in chronic respiratory disease. Longitudinal data from pediatric and adolescent PCD populations have shown no association between changes in BMI over time and changes in spirometric measures, including FEV₁, forced vital capacity (FVC), and forced expiratory flow (FEF) 25-75 in some cases [14]. However, other studies from larger PCD cohorts demonstrate that lower BMI is associated with poorer baseline lung function, FEV₁, and disease progression [15]. These observations illustrate the complexity of the relationship between anthropometric measures and pulmonary function. Parameters such as fat-free mass, for example, may provide a clearer view of disease staging and progression than BMI might not be able to display in some cases [16]. Due to the complexity and variability in predictive parameters used for staging lung function, a more effective approach may involve combining multiple parameters rather than relying on a single measure. Additionally, nonlinear relationships between anthropometric measures and pulmonary function may obscure associations when studied using linear models [17]. In contrast, measures such as skeletal muscle mass and fat-free mass have shown stronger and more consistent associations with lung function in chronic lung disease [16]. Together, these results indicate that a comprehensive assessment of body composition may deliver a more precise characterization of physiologic reserve than BMI alone.

The absence of significant associations should also be interpreted in the context of the limited statistical power of this study (n=16), which reduces the ability to detect moderate correlations between the body composition parameters measured and the patient's lung function. Furthermore, the cohort consisted exclusively of individuals with PCD due to the *RSPH4A* founder variant carrying the c.921+3_921+6delAAGT mutation, a prevalent variant in the Puerto Rican population. While patients with this variant present with PCD features such as neonatal respiratory distress, bronchiectasis, and sinus disease, they may have reduced rates of chronic secretory otitis media and laterality defects, which may be present in other variants of this disease [12]. It is important to mention that, along with these manifestations, around 38-51% of adult patients with PCD have been reported to be in need of a lung transplantation or have been found with severe pulmonary function impairment [18]. This relative clinical stability may attenuate detectable relationships between physiologic parameters. In addition, heterogeneity in nutritional status, inflammatory burden, and metabolic demands may contribute to variation in body composition measures. In chronic lung disease, systemic inflammation, diminished physical activity, poorer nutritional status, and increased resting energy expenditure contribute to muscle wasting and altered fat distribution [13,15]. In lung transplant populations, body composition abnormalities, including sarcopenia, fat, and visceral fat accumulation, among others, have been associated with frailty and adverse clinical outcomes [19]. Frailty, in turn, is strongly linked to increased waitlist mortality, admissions, and poorer rates of post-transplant survival [19]. These findings stress the importance of identifying early markers of impaired physiologic reserve in patients with advanced lung disease.

BIA represents a practical, noninvasive, and portable method for assessing body composition in clinical settings. It allows for repeated measurements with minimal risk and cost. By measuring bioelectrical impedance to estimate fat mass, fat-free mass, and body water compartments, BIA delivers a more detailed characterization of nutritional and physiologic status than BMI alone [13]. These findings suggest that body composition analysis may provide additional physiologic characterization in patients with PCD evaluated in transplant settings, capturing aspects of systemic status not reflected in spirometry alone. In rare diseases such as PCD, in which traditional clinical markers may not fully capture systemic health status, BIA may provide additional descriptive information on physiologic reserve and body composition patterns.

Strengths and limitations

This study has several strengths. First, it uses multi-frequency segmental BIA, enabling a detailed assessment of body composition parameters beyond conventional anthropometric measures, such as BMI. Second, the study focuses on an understudied cohort of patients with PCD caused by the *RSPH4A* founder variant, providing descriptive data in a rare disease population. Third, the inclusion of hydration-related parameters (TBW% and ECW/TBW) provides additional insight into these patients' physiologic reserve that might not be typically captured in routine clinical evaluation.

This study has several limitations. The small sample size reflects the rarity of PCD-*RSPH4A* and limits generalizability, statistical power to detect moderate associations, and precluded sex-stratified analyses. Inflammatory and infection-related markers (e.g., CRP, leukocyte counts, and sputum microbiology) were not available, which may influence interpretation of body composition findings. While BIA is widely used in clinical and research settings, its measurements are influenced by hydration status, recent food intake, physical activity, body positioning, medications, and environmental conditions [13]. Results may limit external reproducibility and cross-device comparability due to the proprietary algorithms used to derive body composition estimates and device-specific reference values. BIA data were limited to the parameters directly provided by the system, precluding the availability of fat-free mass data. Standardized pre-measurement methods and measurement protocols were applied to minimize sources of variability. Finally, the cross-sectional design precludes assessment of longitudinal changes in body composition and their relationship to disease progression or clinical outcomes. Future studies with larger sample sizes and prospective designs are needed to determine whether changes in body composition predict disease progression, transplant candidacy, or clinical outcomes in PCD.

## Conclusions

In this cohort of patients with the PCD-*RSPH4A* variant (n=16), body composition analysis using multi-frequency BIA identified systemic alterations, particularly in hydration status, characterized by reduced TBW% and elevated ECW/TBW relative to individualized targets. Patients aged 25 years or older demonstrated higher numerical variability in BMI and fat percentage. However, a formal statistical comparison of variances was not performed due to the small sample size and limited power. No significant associations were observed between body composition parameters and FEV₁% predicted, a negative finding that may be associated with limited statistical power. The observed heterogeneity in body composition among individuals with similar BMI values suggests that BMI alone may not fully capture physiological differences in this cohort. However, the suggestion that BIA may inform transplant readiness is not supported by the current dataset, as no transplant-related outcomes were assessed. These data should therefore be considered hypothesis-generating rather than clinically applicable.

Overall, these findings are supported by the descriptive BIA data and statistical summaries. Results should be interpreted with caution due to the small sample size and cross-sectional design, which limit inference regarding associations and preclude assessment of longitudinal outcomes. Larger, longitudinal studies are needed to determine whether these parameters are associated with disease progression, lung function decline, or clinically relevant transplant outcomes in patients with PCD due to the *RSPH4A* variant.
